# The outcomes of mini-laparoscopic pyeloplasty in children - brazilian experience

**DOI:** 10.1590/S1677-5538.IBJU.2019.0381

**Published:** 2020-01-10

**Authors:** Cristiane Reis Leonardo, Alexandra Muzzi, José Eduardo Távora, Rodrigo Q. Soares

**Affiliations:** 1 Departamento de Urologia Pediátrica Neocentro Hospital Vila da Serra Nova LimaMG Brasil Departamento de Urologia Pediátrica, Neocentro - Hospital Vila da Serra, Nova Lima, MG, Brasil;; 2 Departamento de Cirurgia Pediátrica Neocentro Hospital Vila da Serra Nova LimaMG Brasil Departamento de Cirurgia Pediátrica, Neocentro - Hospital Vila da Serra, Nova Lima, MG, Brasil;; 3 Departamento de Urologia Neocentro Hospital Vila da Serra Nova LimaMG Brasil Departamento de Urologia, Neocentro - Hospital Vila da Serra, Nova Lima, MG, Brasil

**Keywords:** Child, Laparoscopy, Kidney Pelvis

## Abstract

**Objetive:**

Pelvicureteric junction (PUJ) obstruction is the main cause of hydronephrosis in childhood. Open pyeloplasty has been the gold standard treatment of this condition with success rate above 90%. The role of laparoscopic pyeloplasty (LP) in children is less well defined and has slowly emerged as an alternative procedure. We report outcomes of our initial experience with LP in 38 children from 2 months of age.

**Materials and Methods:**

From June 2015 to December 2017 38 children aged 2-60 months (mean age 1.7 years) underwent LP for correction of PUJ obstruction. The mean pre operative anteroposterior diameter of the renal pelvis (APD) was 43,5mm and all patients had hydronephrosis (APD 21.4-76 mm) and obstructed curve on diuretic renogram. Anderson-Hynes pyeloplasty was the performed technique. Results are reported.

**Results:**

Mean operative time was 107 minutes (70-180) with no conversion to open procedure. Pain control was needed mainly in the first 12hs. Mean hospitalization was 2 days (1-5). There were complications in 5 children not affecting the final outcome. Two patients had a re-obstruction requiring a second procedure with good result. The mean follow up was 18 months (13-36). The mean reduction on the postoperative APD was 41% - p<0,001 (end APD 5 to 41mm). Overall success rate was 94,7%. All children had good cosmetic results.

**Conclusions:**

This is a small series limited by short follow up, however its data suggest that LP has good functional and cosmetic results, not compromising the success of the open procedure, regardless patient age.

## INTRODUCTION

Pelvic-ureteric junction (PUJ) obstruction is a common problem and the main cause of hydronephrosis in childhood. The advent of prenatal ultrasound (US) has brought the diagnosis even earlier, before symptoms occur. Thus, treatment has been proposed at younger ages.

Open pyeloplasty has long been the gold standard treatment of PUJ obstruction in children. The open approach was described by Anderson-Hynes in 1949 with success rates over 90% (1). Laparoscopic pyeloplasty (LP) was first performed in adults in 1993 (2). The approach results are already well established, and it has been the first choice where such technology is available, with advantages of a minimally invasive procedure. The proposed benefits are shorter hospitalization, reduced postoperative pain, early return to daily activities and improved cosmetic appearance, while providing good functional results in a reasonable operative time (3, 4).

The first LP in children was performed in 1995 (5). It has gained popularity for older children. The role of LP in youngers and mainly in infants is less well defined. It has just slowly emerged as safe and an alternative to the open procedure (6).

From the end of 2014 on we started a multidisciplinary program to develop minimally invasive pediatric urology. Since June 2015, all children with PUJ obstruction have been submitted to LP in our institution.

The results of our initial experience with LP in children from 2 months to 5 years old are reported here, addressing pre and postoperative data.

## MATERIALS AND METHODS

From June 2015 to December 2017 38 children undergoing LP due to PUJ obstruction and aged 5 years old or younger were enrolled to this study.

Inclusion criteria were: follow-up of at least 6 months after double J (DJ) stent removal and US after this period of time.

Indications for surgery were worsening hydronephrosis and anteroposterior diameter of the renal pelvis (APD) above 20mm on US, plus obstructed pattern on diuretic renogram (DR)-Tc 99m diethylenetriamine penta-acetic acid (DTPA).

The surgical technique performed was Anderson-Hynes dismembered pyeloplasty.

**Figure 1:**
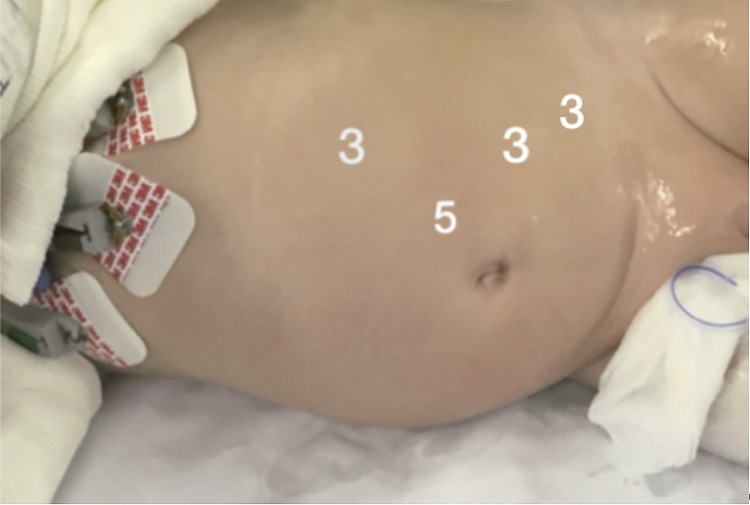
Trocars position - lateral decubitus. Child postioned to left pyeloplasty in a 60-degree lateral decubitus. One 5mm trocar to the telescope placed lateral to the umbilicus on the side of the affected kidney and two 3mm ports placed in a triangular fashion to the telescope port. The last 3mm port is placed in the lower abdomen on the mid clavicular line.

**Figure 2:**
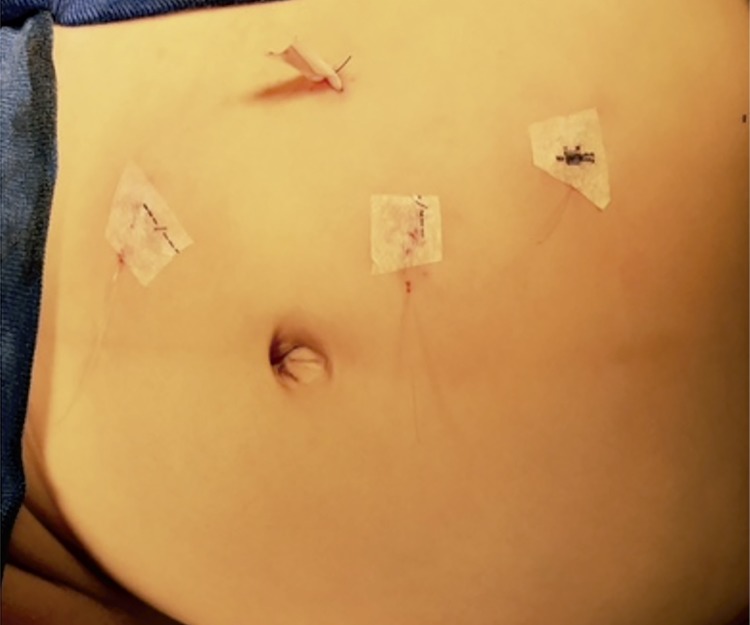
Trocars position - postoperative period. Position of the trocars - right pyeloplasty, one 5mm port to the telescope placed above and lateral to the umbilicus on the side of the affected kidney and three 3mm trocars to the instruments.

LP is performed positioning the child in a 60-degree lateral decubitus with the side to be treated up. Four ports are used: 3 to 3mm instruments (20cm long) and 15mm to the telescope. The telescope is positioned lateral to the umbilicus on the side of the affected kidney. The next 2-3mm ports are placed mid clavicular and anterior axillary lines in a triangular fashion to the telescope port. The last 3mm port is placed in the lower abdomen, also in the mid clavicular line. Warmed pneumoperitoneum is maintained at a mean pressure of 8mmHG. Most of the time, the renal pelvis is easily seen. When necessary, the colon is reflected medially or it is approached trans-mesocolon on the left side. A hitch stitch is placed through the abdominal wall to stabilize the renal pelvis. The PUJ is dismembered and the healthy ureter is spatulated on its lateral aspect. 6.0 (until 2 years old) or 5.0 polyglactin threads (>2 years old) are used in a running suture fashion for pelvi-ureteric anastomosis. Except in a very large APD (>50mm), no attempt is made to trim the dilated pelvis. The anastomosis is performed anterior to anomalous lower pole vessel, when it is present. The DJ stent is placed toward the bladder in an antegrade fashion. A drain is introduced through the lower port. Local bupivacaine is used in all trocar ports. A bladder catheter is left in place during hospitalization (Figures 1 and 2).

Oral intake is started as soon as the patient is recovered from anesthesia.

Pain control is available on patient and or parents demand. Intravenous dipyrone and oral paracetamol are available to usual pain. Morphine is available to non-regular pain.

All patients have been followed by US at 1, 3 and 6 month after DJ removal and thereafter at every 6 months. DR was repeated when renal dilatation persisted on US.

Statistical analysis was performed by statistical software. Data were expressed in mean and range for continuous variables. Students t-test was used to compare pre and postoperative parameters. A p value of <0.05 was considered significant.

## RESULTS

From June 2015 to December 2017 thirty eight children aged 2-60 months (mean 1, 7 years old) underwent LP and were included in this study. All patients were operated on by the same team-2 surgeons (CRL and RSQS).

There were 28 boys and 10 girls. Twenty patients were younger than 1 year old, 6 were between 13 and 24 months and 12 between 25 months to 5 years old. The mean weight was 8.2Kg (4 to 22Kg).

PUJ obstruction was diagnosed by antenatal hydronephrosis in 27 patients, urinary tract infection (ITU) in 6 and abdominal pain in 5 patients.

Comorbidities were present in 6 children: 1 vesicoureteric reflux, 1 horse shoe kidney, 2 chromosome disease, 1 multicystic dysplastic kidney, 1 renal stones. Two patients had previous pyelostomy.

Obstruction was on the left renal pelvis in 20 children (52%).

The mean pre-operative APD was 43.5mm (21.4 to 76mm) on US. Loss of renal cortex was found in 17 patients (68%).

All patients had an obstructed pattern on DR and post furosemide T1/2>20min. Of them, 9 presented split renal function <40% on the affected side Table-1 shows details of the procedure.

**Table 1:** Laparoscopic Pyeloplasty - surgical details.

Pelvis approach	
Colon mobilization	27 (71%)
Transmesocolon	11 (29%)
**Anastomotic thread**	
Vicryl 5.0	12 (32%)
Vicryl 6.0 (<2 years old)	26 (68%)
**Drain**	
Penrose	4
Suction	33
No one	1
Bladder catheter + Double J	38
**Surgical time (min.)**	
Mean	107 min.
Range	70-180 min.
Conversion to open procedure	0

Mean surgical time was 107 minutes (70-180min) from port insertion to port closure. There was no conversion to open procedure. Eleven patients had a lower pole vessel obstructing the PUJ (29%).

Oral intake was started from 40 to 240 min (mean 120min) after the end of the procedure.

Mean time of analgesia requirement was 12hs (0-24hs). Dipyrone was used. No opioid was necessary.

Mean hospitalization was 2 days (1-5 days).

DJ stent was removed in mean after 45 days after the procedure (15-62 days). There were complications in 6 children (15.7%) with no effect on the final outcome. Three children had UTI, one of them needing hospitalization. Two had dislodgement of the DJ stent. One had omental fat exteriorization during drain removal.

Two patients had worsening hydronephrosis and re-obstruction after DJ removal, requiring a second intervention. The second procedure was performed by laparoscopic approach with very good results. The first one had chromosomal disease and abnormal renal vessels. The second one had previous infective stones which were removed at the LP.

Median follow-up was 18 months (from 13 to 36 months).

Mean reduction on the postoperative APD was 41.8% (end APD 5 to 41mm) p <0.001. Three patients had improved, but maintained postoperative hydronephrosis. All of them showed a good washout curve on DTPA. All children are symptoms free. All children had good cosmetic results. Overall success rate was 94.7%, Table-2 shows results according to age.

**Table 2:** Laparoscopic Pyeloplasty - results by age.

N (38)	Age	APD reduction	Surgical time min	Success Rate
20	< 1 year	44,6%	82-180	20/20
6	1-2 years	43%	70-160	6/6
12	3-5 years	38%	98-170	10/12

**N =** number of patients; **APD =** anteroposterior pelvic diameter

All parents of the children signed the informed consent for LP.

## DISCUSSION

PUJ obstruction is a common problem in children. The open dismembered pyeloplasty described by Anderson-‐Hynes has long been the gold standard treatment with success rate above 90%. Although LP has been widely performed in adults, its benefits in infants have been less clear. The minimally invasive approach has slowly emerged as safe and effective alternative to treat PUJ obstruction in children.

By the end of 2014 we started a multidisciplinary program in pediatric urology which comprised adult videourology and pediatric urology teams. Adult experience was already established and after experience was obtained with older children we were able to start operating the younger ones. Since June 2015 all children with PUJ obstruction have been submitted to LP in our institution. We report here the outcomes and details of 38 LP in children aged 2 months to 5 years old.

Minimally invasive surgery has gained the world and its benefits are well known: image magnification, decreased blood loss, lower analgesia requirement, faster recovery, better cosmetic outcome. LP also has another reported benefit. Through laparoscopic view, the PUJ is seen in its real position, in contrast to the open or video assisted procedure which brings the PUJ outside, disrupting its normal anatomy. LP is thought to provide better identification to anomalous vessels and avoid twisting or bad positioning of the ureter (3, 7).

The dismembered LP for treatment of PUJ obstruction in children was first described in 1995 (5). Since then, a few pediatric large series are available in the literature. Many reference centers do not have programs to perform LP in young children-less than 2-3 years old (8). However, while early series had reported anastomotic stenosis in babies (9, 10), subsequent studies demonstrated feasibility irrespective of patient age and weight (6, 7, 11-13).

LP has been thought to be a technically challenging procedure in children. In fact it requires suture training and an experienced laparoscopist (3, 8). There is a learning curve to LP with is far more difficult to pediatric surgeons. Since the beginning of training, pediatric surgeons have smaller and more delicate structures to work with, compared to adult surgeons who find larger structures in their patients. Therefore, limited laparoscopic working space and small ureteral caliber make anastomosis challenging. Also, even in reference centers, the number of pediatric cases in general suitable for laparoscopic procedures in the same period of time is lower to pediatric urology when compared to the adult urology, slowing the learning curve further. Despite this, virtual labs and multidisciplinary practice may be useful to speed the learning curve.

In our institution after having established a per and postoperative protocol, all patients were operated by the same team (2 surgeons CRL and RSQS). Therefore, the surgical steps were redrawn as needed.

Robotic surgery certainly will add technical facilities in pyeloplasty (14, 15). However, the need of larger incisions for larger port placement and no availability of 3mm instruments makes its role in younger children questionable at moment.

Concerning technical details, we use three ports. The third port helps on exposure. Not placing the telescope into umbilical scar brings all the instruments near the target PUJ. This may avoid organs injuries in small spaces as reported even by expert laparoscopists (3, 6).

While operating on babies it is important a full integration of the anesthetic team to laparoscopic procedures at younger ages.

Although most series report longer operative time in LP, (mean 155-240min) (3, 8, 11, 16) we had spent a mean of 107min., which may be near the open procedures time. Previous adult experience, same team, routine and focusing on simplifying every step certainly play a role in the operative time.

DJ stent is inserted by laparoscopic view. A guide wire is placed through a 3mm aspirating tube in an antegrade fashion, saving the cystoscopy time. Those who favor cystoscopy insertion affirm that retrograde DJ insertion avoids stent dislodgement and related complications (8). However, a study of 15 academic European institutions showed that the antegrade fashion provided the lowest complication rate compared with retrograde stent insertion (17).

We had one child whose DJ didn’t reach the bladder. Since then, we focus on urine drops reflowing from the stent as it reaches the bladder. The bladder catheter is inserted at the end of the procedure or otherwise kept closed in the bladder until there.

Surgical site drainage may be a matter of discussion since the postoperative leakage is usually little. Perinephric drain offers the advantage of warning about complications. Postoperative ileus is described by series where drains are avoided (3, 6). We started using a Penrose drain, but moved on to suction drain. Although we had a small omental prolapse, it wasn’t necessary any surgical procedure to deal with. Adequate size Blake® drains may reduce the reported risk (8).

Our children had oral intake soon after anesthetic recovery with very good tolerance. After local bupivacaine injection at the end of the procedure, pain control was on patient and parents demand. It was used in mean during the first 12hs which is a short time when compared with open procedures. No opioids were necessary. Two patients didn’t require any postoperative analgesia.

Hospitalization was in average 2 days. Although a subjective data, patients were noticed to be with more mobility when compared to our experience in open procedures. In a comparative prospective study of open versus laparoscopic pyeloplasty in children, Piaggio et al. observed fewer narcotic need and shorter hospitalization for LP as others (16, 18).

Our study showed very good functional results in 33 children with significant reduction of the hydronephrosis - mean reduction on the postoperative APD was 41.8% (preoperative APD-21 to 76mm and final APD-5 to 41mm) p<0.001. We don’t regularly trim the renal pelvis and the APD reduction found is associated with success rate according to the literature (19).

Four patients underwent DR as they had maintained postoperative hydronephrosis. Despite persistent dilatation, all of them showed a good washout curve on DTPA with no obstructed pattern, ensuring a good result after LP. Two children needed reoperation due to worsening hydronephrosis post operatively. One patient had abnormal chromosomes and disrupted anatomy with intra-‐renal pelvis and anomalous vessels to the kidney. The fail was due to the fact that the pelvic-ureteric anastomosis had been performed above the first anomalous vessels, which seemed to be usual for cases of pyeloplasty. In the second procedure it seemed clear that the anastomosis was again obstructed and needed to be higher and proximal to other anomalous vessels, on a small space available of the abnormal renal pelvis. The other patient had infective stones removed during the first LP. A second laparoscopic procedure was performed in both patients with success, as described in literature (20).

As reported in literature, our study showed no different outcomes related to children’s age or weight (17, 21-23). The overall success rate was 100% in the 20 infants and operative time was compared to laparoscopic procedure for older children.

The overall success rate of 94.7% in this study is similar to open procedure and others reported LP series (24-26).

The cosmetic outcome was very satisfactory, with the 3mm scars barely apparent. In spite of that, the argument of open procedure through small incisions has been still supported by some, who do not consider that even small scars grow with patients development and may cause dissatisfaction later. Concerning pain related to open small incision, it may be underestimated in small children (2, 6, 8, 16).

This study demonstrated functional results as reported to open surgery and benefits of a minimally invasive procedure as described by other series in literature (12, 13 17, 22, 27, 28).

## CONCLUSIONS

Our study is limited by short follow-up and small number of patients, however its data suggests that LP has acceptable percentage of complications, good functional and cosmetic results, not compromising the success of the open procedure, regardless patient age.
